# A comparison of allied healthcare versus no allied healthcare on participation, fatigue, physical functioning and health-related quality of life for patients with persistent complaints after a COVID-19 infection

**DOI:** 10.1080/07853890.2025.2600139

**Published:** 2025-12-10

**Authors:** Ângela Jornada Ben, Anita Natalia Varga, Siméon de Bruijn, Willem Bastiaan Dekker, Cees C. van den Wijngaard, Arie Cornelis Verburg, Thomas Johannes Hoogeboom, Philip van der Wees, Raymond W. J. G. Ostelo, Judith E. Bosmans, Johanna Maria van Dongen

**Affiliations:** ^a^ Department of Health Sciences, Vrije Universiteit Amsterdam, Amsterdam Public Health research institute, Amsterdam, the Netherlands; ^b^ Center for Infectious Disease Control, National Institute for Public Health and the Environment (RIVM), Bilthoven, the Netherlands; ^c^ IQ Health science department, Radboud university medical center, Nijmegen, the Netherlands; ^d^ Department of Epidemiology and Data Science, Amsterdam UMC, Vrije Universiteit Amsterdam, Amsterdam Movement Sciences Research Institute, Amsterdam, The Netherlands

**Keywords:** Long COVID, allied healthcare, rehabilitation, participation, health-related quality of life, physical functioning

## Abstract

**Objective:**

To assess the effectiveness of allied healthcare *versus* no allied healthcare.

**Materials and Methods:**

Data from the ParaCOV cohort (allied healthcare, *n* = 1,451) and the LongCOVID cohort (no allied healthcare/control, *n* = 1427) were analyzed. Average treatment effects (ATEs) between groups were estimated using Targeted Maximum Likelihood Estimation adjusted for age, sex, body mass index, smoking status, comorbidities, and effect outcomes’ baseline values. *A* ≥ 10% between-group difference in improvement from baseline (BTGD) was considered clinically relevant for participation, fatigue, and physical functioning, and ≥0.062 for health-related quality of life.

**Results:**

Patients receiving allied healthcare were older (49.2 vs. 41.2 years), less often female (63.3% vs. 70.1%), had higher BMI (28.2 vs. 26.1), smoked less frequently (5.0% vs. 9.0%), had more comorbidities (49.2% vs. 41.9%), and lower baseline anxiety and depression scores compared to those not receiving allied healthcare. For participation, ATEs after 6 and 12 months were respectively −2.62 (95%CI: −4.39; −0.86) and −1.68 (95%CI: −4.81;1.45), with BTGDs of 4.7% and 1.8% favoring the control. For fatigue, ATEs were 1.72 (95%CI: −0.14; 3.58) and 0.97 (95%CI: −1.48; 3.41), with BTGDs of 6.5% and 3.7% favoring the control. For physical functioning, ATEs were 5.75 (95% CI: 4.42; 7.09) and 6.36 (95%CI: 4.84; 7.88), with BTGDs of 1.4% and 2.2% favoring allied healthcare. For health-related quality of life, ATEs were 0.017 (95%CI: −0.008; 0.0044) and 0.033 (95%CI: 0.011; 0.054).

**Conclusions:**

Patients with persistent complaints after a COVID-19 infection showed significantly lower participation after 6 months, higher health-related quality of life after 12 months, and better physical functioning after 6 and 12 months of allied healthcare, however, BTGDs were not clinically relevant. Study limitations warrant cautious results interpretation.

## KEY MESSAGES

- Although health-related quality of life and physical functioning improved in Long COVID patients, this cannot be definitively attributed to allied healthcare.
- The observed outcome differences between Long COVID patients with and without allied healthcare were not clinically relevant.
- More research is needed for tailored rehabilitation treatments for these patients.

## Introduction

Persistent complaints after a Coronavirus disease 2019 (COVID-19) infection include a variety of symptoms, such as fatigue, arthromyalgia, and memory loss, which is often grouped with other cognitive symptoms (e.g. poor concentration) under the term “brain fog”, for more than 12 weeks after the date of infection [1,2]. These persistent symptoms are also known as Long COVID or Post COVID-19 condition, describing a chronic state that begins within three months of the initial infection and persists for at least two months [3,4]. A large Dutch country-wide survey that cross-sectionally assessed the self-reported prevalence of long-term symptoms after a COVID-19 infection in 2023 found that 3% of adults experienced persistent COVID-19-related complaints [5]. Approximately a quarter of them reported experiencing a severe burden from their complaints, leading to an estimated 100,000 Dutch adults suffering from severe persistent complaints attributed to a COVID-19 infection in 2023 [5]. Evidence has shown that such persistent complaints considerably affect general health and daily living [6–8] and that they are associated with increased healthcare utilization, low productivity at work, and new disability insurance claims [9–12].

Since the start of the pandemic, Dutch adults with persistent complaints after a COVID-19 infection could be referred to allied healthcare by general practitioners or medical specialists for rehabilitation to combat the associated human suffering and economic burden. Allied healthcare is provided in primary care by physiotherapists, exercise therapists, occupational therapists, dietitians, and/or speech, and language therapists. The treatment provided by allied healthcare professionals was temporarily reimbursed for 6 months through the Dutch Basic Health Insurance Package, and if recovery was not achieved, treatment could be extended for an additional 6 months, under medical referral [13]. Between 2020 and 2022, approximately 170,000 Dutch adults with persistent complaints after a COVID-19 infection received funding for allied healthcare through this program [14]. However, this reimbursement was conditional and would only be continued if the effectiveness of allied healthcare for persistent complaints after a COVID-19 infection could be shown [13].

To assess recovery trajectories and costs of patients receiving allied healthcare for treating COVID-19-related complaints, patients were invited to participate in a cohort to assess their progress after 3, 6, 9, and 12 months in the Dutch “Primary Allied Health Care in Patients Recovering From COVID-19” study (i.e. the ParaCOV study) [15]. Results of the ParaCOV study showed that the health status of patients with persistent complaints after a COVID-19 infection improved during the 12 months after receiving allied healthcare, but limitations in daily activities and participation remained [16,17]. Due to the lack of a control group, however, no conclusions could be drawn regarding the effectiveness of allied healthcare for patients with persistent COVID-19-related complaints. This study therefore aims to assess the effectiveness of ‘allied healthcare’ *versus* ‘no allied healthcare’, by matching the ParaCOV cohort data to data of a cohort of patients with persistent complaints after a COVID-19 infection who did not receive allied healthcare from the LongCOVID cohort of the Dutch National Institute for Public Health and the Environment (RIVM) [15]. The comparative effectiveness of ‘allied healthcare’ *versus* ‘no allied healthcare’ was assessed for participation, health-related quality of life, fatigue, and physical functioning.

## Materials and methods

### Study design and population

This study was conducted using cohort data from the ParaCOV study [15] and the LongCOVID study [18] and it adheres to the Declaration of Helsinki. Both cohort studies, on which the current study is based, were exempted from ethics approval for human subjects research by their respective medical ethics committees (Radboudumc: 2020–7278, Utrecht Medical Ethics Committee: 21-124/C) as both studies did not involve medical interventions or impose behavioral requirements on participants. Written informed consent was obtained from all participants in both studies.

The ParaCOV study was initiated to evaluate the longitudinal recovery trajectories and related costs of adult patients (≥ 18 years) who visited an allied healthcare provider to manage any form of persistent complaints after a self-reported COVID-19 infection [15–17,19]. The term “persistent complaints” was therefore used to reflect that anyone with self-reported persistent symptoms following a self-reported COVID-19 infection was eligible to receive allied healthcare under the Dutch Basic Health Insurance Package at that time. Because both the infection and symptoms were self-reported and not clinically verified, we could not confirm how long participants had been experiencing persistent symptoms following a COVID-19 infection at the time of enrollment. Besides allied healthcare, patients also received usual care (i.e. mental health care such as psychologist and social work consultations and complementary care such as homeopathy, osteopathy, Reiki, Yoga, etc.). Patients were enrolled between March 29 and June 19, 2021, and were followed for 12 months [15]. The ParaCOV study was exempted from ethics approval for human subjects research by the medical ethics committee of Radboudumc (registration number 2020–7278) and was registered in the clinicaltrials.gov registry (NCT04735744) [15]. Informed consent was obtained from all patients prior to enrolment in the study [15]. Based on the inclusion period, the population of the ParaCOV study most likely had the wild-type or Alpha variant of COVID-19 [16,20]. In the present study, the ParaCOV cohort was considered the ‘allied healthcare’ group, including primary care provided by any of the following healthcare professionals: physiotherapists, exercise therapists, occupational therapists, dietitians, and/or speech, and language therapists. As shown in a previous publication, participants primarily made use of physiotherapy and occupational therapy as forms of allied healthcare [17].

The LongCOVID study was initiated to assess the prevalence and severity of any form of persisting symptoms with a duration of at least 2 months in individuals infected by COVID-19 compared with individuals who were not infected [18]. Since May 2021, the LongCOVID study has collected retrospective and prospective data from participants aged ≥5 years with self-reported COVID-19 infection [18]. Among them, some participants received usual care (i.e. mental health care and complementary care) or/and allied healthcare (either for treating persistent complaints related to or unrelated to a COVID-19 infection), whereas others did not [18]. The Long COVID study was exempted from ethics approval for human subjects research by the Utrecht Medical Ethics Committee (METC protocol number 21-124/C) [18]. Informed consent was obtained from all participants prior to enrolment in the study [18]. In the present study, the eligible LongCOVID cohort was considered the ‘no allied healthcare’ group (interchangeably referred to as the control group).

### Inclusion criteria

The ParaCOV study included adults referred to primary care allied health services by a general practitioner or medical specialist for treating self-reported persistent COVID-19-related complaints, regardless infection test confirmation [15–17,19]. The LongCOVID study included children (aged 5–17) and adults (≥ 18 years) participants who self-reported persistent symptoms associated with COVID-19 infection with or without infection test confirmation [18].

**Figure 1.**
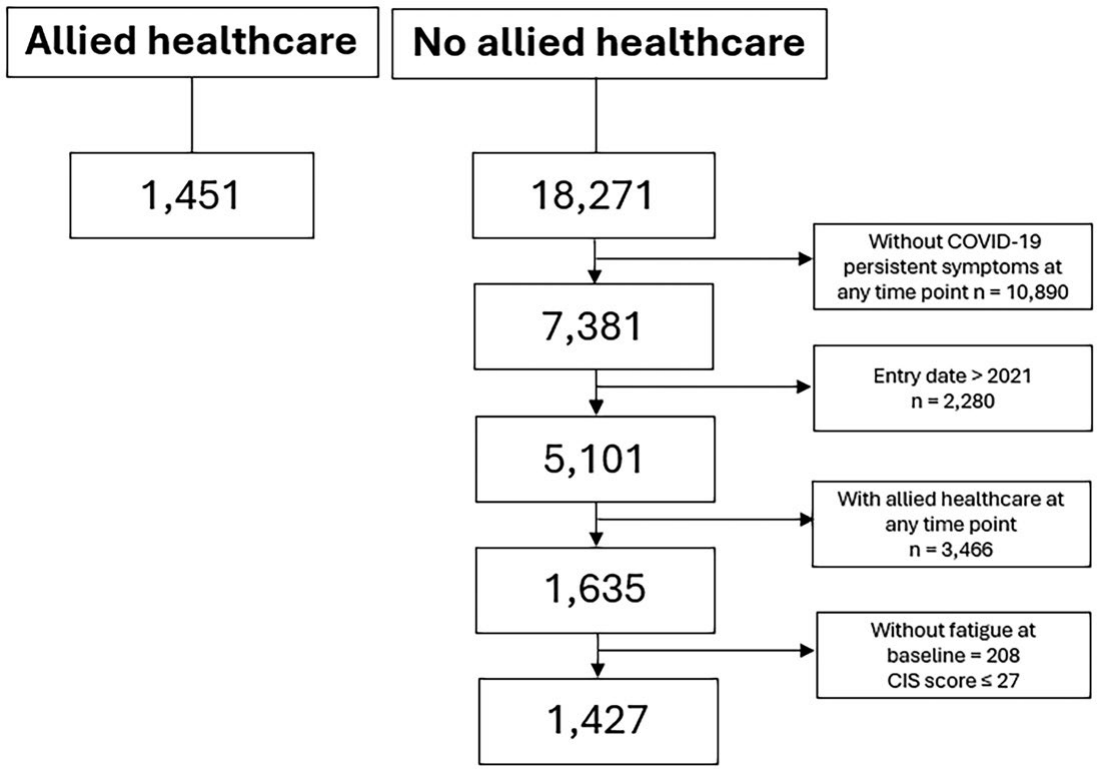
Flowchart showing the study population from the ParaCOV cohort and the LongCOVID cohort according to eligibility criteria. CIS = Checklist Individual Strength. The mean CIS score ≤27 was used as a cut-off point to ensure comparability between both cohorts in terms of fatigue at baseline. NB: Please note that we only received LongCOVID data of adult people with a self-reported positive COVID-19 test.

Additional eligibility criteria were applied to the LongCOVID cohort prior to the analysis of the data to ensure comparability to the ParaCOV cohort: 1) presence of self-reported persistent COVID-19-related complaints during the full study period (yes/no); 2) not receiving any type of allied healthcare during the full study period (related or unrelated to COVID-19); 3) entry date in the study in 2021; 4) presence of self-reported fatigue at baseline; and 5) age ≥18 years at baseline (Figure 1). To assess the presence of fatigue at baseline, the Checklist Individual Strength (CIS) fatigue score of ≥27 was used [21]. This CIS score represents the minimum score reported by patients with chronic fatigue in its validation study [21]. Moreover, before the main analysis, we assessed whether the balance between the baseline values for the effect outcome of both cohorts would improve when using a different CIS score, i.e. ≥23 (Appendix a) and ≥35 (Appendix b). As this was not the case, the CIS score of ≥27 was used to select participants for this comparative effectiveness study.

### Comparators

In the current study, ParaCOV cohort patients who received allied healthcare, i.e. the ‘allied healthcare’ group, were compared to LongCOVID cohort participants who did not receive allied healthcare, i.e. the ‘no allied healthcare’ group. Allied healthcare included standard treatments provided by one or more allied health professionals (i.e. physiotherapists, exercise therapists, occupational therapists, dietitians, and/or speech and language therapists) based on guidelines for COVID-19 published by the professional bodies of the allied health professionals as available at the start of the cohort [15].

### Baseline characteristics

Baseline characteristics that were measured and could be harmonized between both cohorts included age (years), sex (male/female), body mass index (BMI) in kg/m^2^, smoking status (never/former, or current), presence of comorbidities (none or ≥1), and possible anxiety and depression. Comorbidities included self-reported hypertension, diabetes mellitus, chronic obstructive pulmonary disease, kidney disease, liver disease, immune disease, oncological disease/malignancies, and chronic neuromuscular disorders in both cohorts. Possible anxiety/depression was measured by the 14-item Hospital Anxiety and Depression Scale (HADS) in both cohorts [22]. The HADS questionnaire is divided into two subscales including 7 items for anxiety (HADS-A) and 7 items for depression (HADS-D), both with 4 response levels ranging from 0 (i.e. often) to 4 (i.e. very rarely). The total score for each subscale is the sum of the responses to each question [22].

### Effect outcomes

Effect outcomes included participation (i.e. the primary outcome of the ParaCOV study [15,17]), health-related quality of life, fatigue, and physical functioning at baseline, 6- and 12-month follow-up. The choice of effect outcomes was based on the literature [23–25], the expertise of the investigators, the possibility of harmonizing outcome measures between cohorts, and a focus group with patient representatives and representatives of professional allied healthcare organizations.

Participation was measured by the Utrecht Scale for Evaluation of Rehabilitation-Participation (USER-P) subscale restrictions [26] in the ParaCOV study. The USER-P restrictions subscale includes 11 questions scored on a 4-point Likert scale with minimum and maximum scores of 11 and 44, respectively [26]. This instrument captures a person’s involvement in daily life activities through self-reported frequency, perceived restrictions, and satisfaction with participation. In the LongCOVID study, it was measured by the Short Form (12) Health Survey (SF-12) Role Physical Limitations questions (i.e. 2 questions, range sum of scores: 2–10) [27]. Total scores for both questionnaires were calculated as the sum of responses and subsequently rescaled to range between 0 and 100, with high scores representing few experienced restrictions, i.e. high participation.

Health-related quality of life was measured by the 5-level EQ-5D version (EQ-5D-5L) in both studies. The EQ-5D-5L includes five dimensions of quality of life (i.e. mobility, self-care, usual activities, pain/discomfort, and anxiety/depression) with five response levels (i.e. no problems, slight problems, moderate problems, severe problems, unable to/extreme problems) [28]. The participants’ health states obtained from EQ-5D-5L responses were converted into utility values using the Dutch tariff [29]. Utility values represent a participant’s preference for a certain health state on a scale anchored at 0 (equal to death) and 1 (equal to optimal health) [29]. The Dutch EQ-5D-5L value set ranges from −0.446 to 1[29].

Fatigue was measured by the Fatigue Severity Scale (FSS) [30] in the ParaCOV study, while in the LongCOVID study, it was measured by the Checklist Individual Strength Fatigue (CIS) [21]. The FSS questionnaire includes 9 questions scored on a 7-point Likert scale, with minimum and maximum scores of 9 and 63, respectively. The CIS-Fatigue questionnaire includes 8 questions scored on a 7-point Likert scale, with minimum and maximum scores of 8 and 56, respectively [21]. Total scores for both questionnaires were calculated as the sum of responses and subsequently rescaled to range between 0 and 100, with high scores representing more fatigue.

Physical functioning was measured by the Patient-Reported Outcomes Measurement Information System Physical Functioning Short Form 10b (PROMIS-PF-10b) [31] in the ParaCOV study. The PROMIS-PF-10b includes 10 questions scored on a 5-point Likert scale, with minimum and maximum raw scores of 10 and 50, respectively [31]. In the LongCOVID study, functioning was measured by the SF-12 Physical Component Summary (i.e. 6 questions, range sum of scores: 6–26) [32]. Raw scores of the PROMIS-PF-10b are typically converted to T-scores, but for comparing outcomes in the current study, total scores for both questionnaires were calculated as the sum of responses and subsequently rescaled to range between 0 and 100, with high scores representing better physical functioning. Evidence has shown that PROMIS physical functioning has a strong correlation with SF-12 Physical Component Summary [33].

### Data analysis

An exploratory descriptive analysis of complete and missing data was performed in both cohorts. Missing data were imputed for each cohort separately using Multivariate Imputation by Chained Equations (MICE) with Predictive Mean Matching (PMM) [34] using SPSS statistics 25 (IBM). Imputation models included baseline characteristics and follow-up data on all effect outcomes. Five imputed datasets were created. The effectiveness analysis was performed per imputed dataset as outlined below, after which results were pooled using Rubin’s rules [34].

Baseline characteristics were described as n (%) for categorical variables and mean (standard error, SE) for continuous variables based on imputed data and tested using t-test and Chi-squared tests (p-value <0.05). Baseline characteristics were compared between ParaCOV cohort patients (i.e. ‘allied healthcare’ cohort) and LongCOVID cohort participants (i.e. ‘no allied healthcare’ cohort). This comparison was conducted to check whether there were systematic statistically significant differences at baseline between the ParaCOV and the LongCOVID cohorts that may explain the differences between study populations besides allied healthcare.

Baseline characteristics were additionally compared between ParaCOV cohort patients (i.e. ‘allied healthcare’ cohort) and LongCOVID cohort participants who did receive allied healthcare (further referred to as ‘LongCOVID with allied healthcare’ cohort). This additional comparison was conducted to check whether there were systematic statistically significant differences between patients receiving allied healthcare in the ParaCOV cohort and the LongCOVID cohort.

Targeted Maximum Likelihood Estimation (TMLE) was used to estimate the average treatment effect (ATE) between groups (i.e. the main analysis) [35]. TMLE estimates the conditional mean of an outcome given the treatment group and possible confounders while taking into account the conditional probability of being treated given the observed confounders [35]. TMLE is considered a doubly robust method because it can produce consistent estimates of the parameters even if either the treatment uptake or outcome model is misspecified. The TMLE was implemented in four steps: 1) estimate the conditional mean of the effect outcomes given the treatment group and confounders (i.e. age, sex, BMI, smoking status, presence of comorbidities, anxiety/depression at baseline, and effect outcomes’ baseline values) by fitting a regression model to predict the outcome. For this purpose, a flexible machine learning model (i.e. a Super Learner) was used to allow for more flexibility and therefore relax distributional assumptions; 2) estimate the propensity score using the same set of confounders as in step 1, for which Super Learner was used; 3) update the initial estimates with the additional information obtained from the inverse propensity score; and 4) average the difference between these updated expected predictions to construct the ATE, and obtain standard errors based on the estimator’s influence curve. Density plots were used to assess the similarity of groups after matching (i.e. to assess propensity score overlap) [36]. The larger the overlap, the more similar groups are based on their propensity scores [36]. Both unadjusted and adjusted estimates were presented. Adjusted estimates were derived using the TMLE method described above, whereas unadjusted estimates were based on an unadjusted linear regression model. Differences between ‘allied healthcare’ and ‘no allied healthcare’ in effect outcome estimates were considered statistically significant when the 95% confidence interval did not contain the null value. For health-related quality of life, we assumed a between-group difference of ≥0.062 to be clinically relevant [37]. In the absence of established minimally clinically important differences for participation, fatigue, and physical functioning, we made a considered assumption that a between-group difference in the percentage of improvement from baseline of ≥10% would be clinically relevant. To estimate the between-group percentage of improvement from baseline, the relative change from the baseline in participation, fatigue, and physical functioning scores at 6 and 12 months of allied healthcare were calculated per patient (Formula 1).

Formula 1 $$\Delta =\frac{\text{outcome}\;\text{scor}e_{t}-\text{outcome}\;\text{scor}e_{b}}{\text{outcome}\;\text{scor}e_{b}}$$

where Δ represents the relative change from baseline, ${\text{outcome score}}_{t}$ represents the outcome score at the time t (i.e. 6 months or 12 months after allied healthcare); and ${\text{outcome score}}_{b}$ represents the outcome score at baseline b.

Subsequently, the resulting relative changes from baseline were included as the outcome variable in the analysis model to adjust the between-group differences for possible confounders. Obtained adjusted between-group differences in improvement from baseline were multiplied by 100 to get the between-group difference in the percentage of improvement from baseline.

### Sensitivity analysis

Two additional sensitivity analyses were conducted to check the robustness of the main analysis results. First, a complete-case analysis (SA1), including data of participants with completely observed data only, was conducted to assess the study results’ robustness to the use of multiple imputation for handling missing data. Second, a sensitivity analysis (SA2) was conducted comparing the ParaCOV cohort participants with LongCOVID participants who received allied healthcare unrelated to persistent complaints after a COVID-19 infection and those who did not receive allied healthcare. SA2 was conducted to evaluate the robustness of our main findings by determining whether the possible inclusion of LongCOVID participants who received allied healthcare for reasons unrelated to COVID-19 had biased the results.

## Results

### Participant selection and characteristics

Initially, the ParaCOV cohort and LongCOVID cohort consisted of 1,451 and 18,271 participants, respectively. After applying the eligibility criteria for the current study, 1,427 participants with persistent complaints after a COVID-19 infection who did not receive any sort of allied healthcare from the LongCOVID study were included in the main analysis (Figure 1). The proportion of missing data in baseline characteristics and outcomes at baseline in both cohorts varied between 0% and 28.4%, while the proportion of missing data on the effect outcomes at follow-up ranged between 35.2% and 76.9% (Appendix c).

Patients who received allied healthcare were statistically significantly older (49.2 *versus* 41.2 years), less often female (63.3% *versus* 70.1%), had a higher BMI (28.2 *versus* 26.1), had a lower proportion of smoking behavior (5.0% *versus* 9.0%), more frequently had comorbidities (49.2% *versus* 41.9%) and had lower anxiety and depression scores at baseline compared to participants of the LongCOVID study who did not receive allied healthcare (Table 1). Both groups had similar levels of fatigue and physical functioning, at baseline, whereas patients who received allied healthcare had statistically significantly lower participation and health-related quality of life compared to participants who did not receive allied healthcare (Table 1). The frequency with which people smoked and/or had comorbidities was similar between the ‘allied healthcare’ and the ‘LongCOVID with allied healthcare’ group, whereas both groups statistically significantly differed in the other baseline characteristics (i.e. age, sex, BMI, anxiety, depression, fatigue, physical functioning, participation, and health-related quality of life, appendix d).

**Table 1.** Baseline characteristics of participants.

Baseline characteristic	Allied healthcare *n = 1,451* (ParaCOV)	No allied healthcare *n = 1,427* (LongCOVID)	*p*-value
Age, mean (SE)	49.2 (0.4)	41.4 (0.4)	0.001
Female, n (%)	917 (63.2)	1001 (70.1)	0.001
BMI, mean (SE)	28.2 (0.2)	26.1 (0.1)	0.001
Smoking status			
Never/ former	1361 (95.0)	1298 (91.0)	0.001
Current	71 (5.0)	129 (9.0)	
Comorbidities, n (%)			
None	727 (50.8)	829 (58.1)	0.001
≥1 comorbidity	705 (49.2)	598 (41.9)	
HADS-A, mean (SE)	7.0 (0.1)	11.9 (0.07)	0.001
HADS-D, mean (SE)	7.3 (0.1)	9.3 (0.05)	0.001
Participation, mean (SE)	66.0 (0.5)	68.3 (0.8)	0.020
Health-related quality of life, mean (SE)	0.629 (0.006)	0.727 (0.005)	0.001
Fatigue, mean (SE)	76.9 (0.4)	75.9 (0.4)	0.113
Physical functioning, mean (SE)	59.6 (0.5)	61.0 (0.6)	0.078

SE = standard error. n = number of participants. % = proportion. BMI = Body Mass Index. Comorbidities included hypertension, diabetes mellitus, chronic obstructive pulmonary disease, kidney disease, liver disease, immune disease, oncological disease/malignancies, and chronic neuromuscular disorders. HADS-A = Hospital Anxiety and Depression Scale possible anxiety. HADS-D = Hospital Anxiety and Depression Scale possible depression. HADS scores ≥ 11 indicate a high probability of anxiety or depression. Participation was measured by the Utrecht Scale for Evaluation of Rehabilitation- Participation (USER-P) subscale restrictions in the ParaCOV study, while in the LongCOVID study, it was measured by the SF-12 Role Physical Limitation questions, both rescaled to 0–100, high scores represent high participation. Health-related quality of life was measured by the EQ-5D-5L; the Dutch EQ-5D-5L tariffs range from −0.446 to 1 (full health). Fatigue was measured by the Fatigue Severity Scale (FSS) in the ParaCOV study while in the LongCOVID study, it was measured by the Checklist Individual Strength (CIS), both rescaled to 0–100, high scores represent more fatigue. Physical functioning was measured by the Patient-Reported Outcomes Measurement Information System Physical Functioning Short Form 10b (PROMIS-PF-10b) in the ParaCOV study while in the LongCOVID study, measured by the SF-12 Physical Component Summary, both rescaled to 0–100, high scores represent better physical functioning.

### Effectiveness

**Figure 2.**
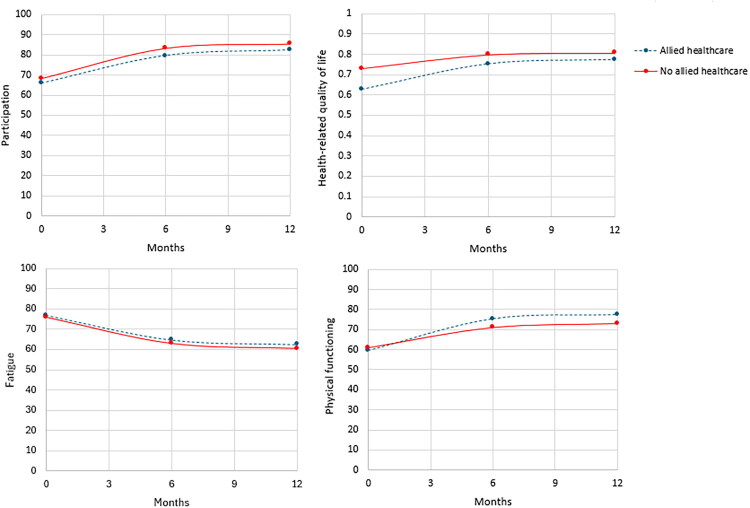
Line plots showing the changes in the mean scores of participation, health-related quality of life, fatigue, and physical functioning over time for patients who received allied healthcare and participants who did not receive allied healthcare, before Targeted Maximum Likelihood Estimation adjustments. Months are determined as months since baseline.

On average, participation, health-related quality of life, fatigue, and physical functioning scores improved from baseline to 6 months and from 6 months to 12 months among patients who received allied healthcare as well as among those who did not (Figure 2).

The *unadjusted analyses* showed statistically significant lower participation scores after 6 months, lower health-related quality of life after 6 and 12 months, no statistical differences in fatigue between groups, and statistically significantly better physical functioning after 6 and 12 months of allied healthcare compared to no allied healthcare (Table 2).

**Table 2.** Effect outcomes by group and differences at 6- and 12-month follow-up.

	Before adjustment			After adjustment	Clinical relevance
Effect outcomes, mean (SE)	Allied healthcare	No allied healthcare	Difference^¥^ (95% CI)	Difference (95% CI)	Between-group % improvement from baseline
**Main analysis**	***n = 1,451***	***n = 1,427***			
Participation at 6 months	79.56 (0.5)	83.25 (0.8)	−3.69 (−5.48; −1.89)	**−2.62 (−4.39; −0.86)**	**−4.7%**
Participation at 12 months	82.29 (0.5)	85.40 (1.4)	−3.11 (−6.3; 0.12)	−1.68 (−4.81; 1.45)	−1.8%
Health-related quality of life at 6 months	0.753 (0.005)	0.798 (0.006)	−0.045 (−0.060; −0.029)	0.017 (−0.008; 0.044)	NA
Health-related quality of life at 12 months	0.774 (0.006)	0.807 (0.006)	−0.033 (−0.051; −0.015)	**0.033 (0.011; 0.054)**	NA
Fatigue at 6 months	64.74 (0.6)	62.87 (0.8)	1.86 (−0.25; 4.0)	1.72 (−0.14; 3.58)	−6.5^§^
Fatigue at 12 months	62.47 (0.7)	60.43 (0.6)	2.04 (−0.49; 4.6)	0.97 (−1.48; 3.41)	−3.7^§^
Physical functioning at 6 months	75.28 (0.7)	71.03 (0.6)	4.25 (2.78; 5.73)	**5.75 (4.42; 7.09)**	**1.4**
Physical functioning at 12 months	77.33 (0.6)	73.00 (0.4)	4.33 (2.34; 6.31)	**6.36 (4.84; 7.88)**	**2.2**
**SA1**	***n = 419***	***n = 262***			
Participation at 6 months	77.52 (1.0)	82.30 (1.4)	−4.78 (−8.10; −1.45)	−3.08 (−6.45; 0.29)	−3.5
Participation at 12 months	81.00 (1.0)	85.35 (1.2)	−4.35 (−7.56; −1.15)	−3.20 (−6.38; −0.02)	−4.7
Health-related quality of life at 6 months	0.736 (0.010)	0.797 (0.012)	−0.061 (−0.093; −0.030)	0.007 (−0.033; 0.047)	NA
Health-related quality of life at 12 months	0.770 (0.010)	0.806 (0.012)	−0.036 (−0.068; −0.004)	**0.036 (0.001; 0.073)**	NA
Fatigue at 6 months	67.46 (1.0)	60.18 (1.4)	7.28 (3.93; 10.63)	**4.25 (1.05; 7.44)**	**−6.1** ^§^
Fatigue at 12 months	64.56 (1.1)	58.82 (1.4)	5.74 (2.16; 9.33)	2.92 (−0.42; 6.28)	−4.1^§^
Physical functioning at 6 months	72.85 (1.0)	70.71 (1.1)	2.14 (−0.92; 5.20)	**4.78 (2.42; 7.14)**	**0.51**
Physical functioning at 12 months	75.46 (1.0)	72.63 (1.2)	2.83 (−0.30; 5.97)	**5.29 (2.79; 7.79)**	**2.1**
**SA2**	***n = 1,451***	***n = 1,892***			
Participation at 6 months	79.56 (0.5)	81.91 (0.9)	−2.35 (−4.23; −0.46)	−1.30 (−3.20; 0.60)	2.7
Participation at 12 months	82.29 (0.5)	82.86 (0.7)	−0.57 (−2.73; 1.59)	0.16 (−1.52; 1.85)	6.5
Health-related quality of life at 6 months	0.753 (0.005)	0.790 (0.005)	−0.037 (−0.052; −0.021)	0.007 (−0.007; 0.022)	NA
Health-related quality of life at 12 months	0.774 (0.006)	0.789 (0.005)	−0.015 (−0.029; 0.007)	**0.029 (0.013; 0.046)**	NA
Fatigue at 6 months	64.74 (0.6)	62.90 (0.7)	1.84 (−0.12; 3.80)	1.48 (−0.26; 3.23)	−4.5^§^
Fatigue at 12 months	62.47 (0.7)	61.03 (0.9)	1.44 (−1.25; 4.13)	0.80 (−1.59; 3.20)	−2.0^§^
Physical functioning at 6 months	75.28 (0.7)	70.41 (0.4)	4.87 (3.23; 6.53)	**5.87 (4.28; 7.45)**	**1.1**
Physical functioning at 12 months	77.33 (0.6)	71.50 (0.8)	5.83 (3.63; 8.03)	**7.15 (5.64; 8.66)**	**3.7**

n = number of participants. SE = standard error. Effect outcomes on a (0–100) scale, except for health-related quality of life (0–1). **^¥^**Differences between groups before adjustment were based on an unadjusted linear regression. Differences after adjustment included age, sex, body mass index, smoking status, comorbidities, and values of the effect outcomes values at baseline. SA1 = sensitivity analysis including only complete observed data. SA2 = sensitivity analysis including participants who did not receive allied healthcare + those who received allied healthcare but unrelated to persistent complaints after a COVID-19 infection. ^§^ = for fatigue outcome, between-group % improvement from baseline was multiplied by −1 for easy interpretation, i.e. the % of improvement from baseline was in favor of the control group. NA = between-group differences in improvement were not assessed for health-related quality of life as a difference of ≥0.062 between groups was assumed to be clinically relevant.

Effect differences between groups in the TMLE were adjusted for age, sex, BMI, smoking status, comorbidities, and the respective effect outcomes’ baseline values. These baseline characteristics resulted in the best propensity score overlap ensuring comparability between cohorts (appendix e). Similar to *unadjusted analyses*, *adjusted analyses* showed statistically significant lower participation scores after 6 months, no statistical differences in fatigue between groups, and statistically significantly better physical functioning after 6 and 12 months of allied healthcare compared to no allied healthcare. In contrast to the *unadjusted analysis*, the *adjusted analysis* showed no statistically significant difference in health-related quality of life at 6 months, but significantly higher health-related quality of life after 12 months of allied healthcare compared to no allied healthcare.

Health-related quality of life improvements were in favor of the allied healthcare group but below the minimally clinically relevant threshold of ≥0.062. For participation, fatigue, and physical functioning, the estimated between-group differences in the percentage of improvement from baseline were all below the clinically relevant threshold of ≥10% (Table 2, column 6).

### Sensitivity analysis

The results of SA1 and SA2 were roughly similar to those of the main analysis (Table 2), except that in SA2 – where LongCOVID participants who received allied healthcare unrelated to persistent complaints after a COVID-19 infection were included in the no allied healthcare group – between-group differences in the percentage of improvement from baseline for participation shifted in favor of the allied healthcare group, instead of in favor of the control group as seen in the main analysis. Despite this shift, between-group differences in the percentage of improvement from baseline remained below the ≥10% threshold (Table 2). A flowchart of participants selected for the SA2 (appendix f) and their baseline characteristics are described in appendix g.

## Discussion

### Main findings

This study compared allied healthcare to no allied healthcare in patients with persistent complaints after COVID-19. Participation was lower at 6 months, health-related quality of life was higher at 12 months, and physical functioning improved at both time points in the allied healthcare group, while fatigue showed no significant differences. However, none of the between-group differences were clinically relevant. Sensitivity analyses supported these findings, though improvement in participation slightly favored the allied healthcare group – but still not reached clinical relevance.

### Explanation of findings and comparison with the literature

A comparison of our results with the literature is hampered by the fact that healthcare for people with persistent complaints after a COVID-19 infection is provided and reimbursed differently across countries. For example, the National Health Service (NHS), in the United Kingdom provides multidisciplinary rehabilitation for persistent COVID-19-related complaints including physical, but also cognitive, and psychological treatment [38]. In addition, there is a lack of studies evaluating the effectiveness of allied healthcare for persistent COVID-19-related complaints. Nonetheless, a few randomized trials investigated the effectiveness of specific rehabilitation treatments for persistent complaints after a COVID-19 infection, such as respiratory rehabilitation. To illustrate, a systematic review of 6 low-quality randomized clinical trials (RCT) suggests that respiratory exercises (e.g. inspiratory muscle training) for people with persistent respiratory complaints and fatigue may positively impact their recovery after 6 weeks of rehabilitation and improve their health-related quality of life compared to usual care [39]. Jimeno-Almazán et al. 2022 [40] showed that an 8-week tailored multicomponent exercise program for patients with persistent complaints after a COVID-19 infection improved fatigue (FSS: −31.2% versus −1.1%, *p* = 0.02) and health-related quality of life (SF-12 physical activity domain: 41.5% *versus* 6.5%, *p* = 0.003) after 8 weeks of follow-up compared to usual care. Philip et al. [41] showed that a 6-week online breathing and well-being program developed for people with Long COVID experiencing breathlessness improved the mental health component of health-related quality of life measured by the Short Form (36) Health Survey – SF-36 (2.42, 95%CI 0.03 to 4.80); *p* = 0·047), but not the physical health component (0.60 95%CI −1,33 to 2.52; *p* = 0·54) after 1-month follow-up compared to usual care.

A recent pragmatic RCT of McGregor et al. [42], not included in the systematic review, showed that online weekly home-based, live, supervised, group exercise and psychological support sessions for 8 weeks improved fatigue (PROMIS: 2.50, 95%CI 1.19 to 3.81), *p* < 0.001), and health-related quality of life (EQ-5D-5L: 0.03, 95%CI 0.01 to 0.05), *p* = 0.02) at 3 and 12 months compared with usual care. Despite several methodological differences between our study and the studies conducted by Jimeno-Almazán et al. Philip et al. and McGregor et al. (i.e. design, population, intervention, follow-up, and outcome measures) results were consistent in the sense that health-related quality of life improved with treatment compared to usual care.

It could be argued that the considered threshold for clinical relevance for between-group differences in improvement from baseline (i.e. *a* ≥ 10%) might not have been optimal, because there is some evidence suggesting that a 20% between-group difference in improvement with physiotherapy (a type of allied healthcare) compared to no treatment is considered clinically relevant by patients with musculoskeletal pain [43,44]. However, we considered *a* ≥ 10% between-group difference in improvement from baseline a reasonable starting point for the interpretation of the clinical relevance given the heterogeneous nature of LongCOVID and the uncertainty about the most appropriate type of allied healthcare for a condition as diverse as Long COVID. Furthermore, it is important to take note of the fact that the use of a stricter threshold would not have changed our overall conclusion.

### Strengths and limitations

This is the first study assessing the comparative effectiveness of allied healthcare *versus* no allied healthcare among Dutch people with persistent complaints after a COVID-19 infection. The findings of this study are important to inform policymakers and healthcare professionals about the effectiveness of allied healthcare for people coping with persistent complaints after a COVID-19 infection [13]. A strength of the study is that advanced statistical techniques were used to deal with its non-randomized nature (i.e. TMLE) [45]. Despite outcomes propensity scores overlap being relatively good, our results might still be confounded by unobserved factors that could not be adjusted for in the analyses (i.e. unmeasured confounding) [46]. Such factors might include infection-related factors (e.g. time since infection, severity), psychological factors (e.g. personality traits, coping mechanisms, social support), lifestyle factors (e.g. physical activity, nutrition), and physiological factors (e.g. exercise intolerance). Therefore, the differences in effects observed between the groups over time may reflect not only the intervention but also unmeasured confounding between the cohorts.

Both cohorts relied on self-reported data without clinical evaluation and standardized diagnosis. As a result, the chronicity of COVID-19-related complaints could not be determined and it was not possible to control for the full range of Long COVID phenotypes or severity in our analyses. It is realistic to assume that these factors have an impact on the clinical outcomes of participants and thus the results of this study. For example, patients who received allied healthcare might have had different ideas about its effectiveness and/or a different response to allied healthcare compared to those who did not. In combination with the fact that all outcomes were self-reported and not clinically tested, this may have led to an over or underestimation of the effect of allied healthcare. At the time of the study, participants mostly received physiotherapy and occupational and little was known about which individuals would benefit most from allied healthcare, a gap that may now be better understood.

Moreover, to minimize bias and improve comparability between groups, patients who did not experience fatigue at baseline were excluded. We chose to focus on fatigue because it is consistently reported as the most prevalent symptom of Long COVID [2]. While we acknowledge that other symptoms may also influence health-related quality of life and the (cost-)effectiveness of allied health interventions, focusing on fatigue allowed us to create a more homogeneous study population and reduce confounding – an important consideration given the non-randomized design. It is also important to note that both cohorts measured fatigue, physical functioning, and participation using different self-reported questionnaires. We used a generic approach for comparing these outcome measures by recalculating scores to a 0–100 score for semantically related questions due to the lack of standardized mapping methods to link one questionnaire to another. Even though the questionnaires used in both cohorts set out to measure a similar construct, this may still have affected the reliability of our results. However, we do not expect this to have severely biased our results, as previous studies have shown moderate correlations between the USER-P and the SF-12 (*r* = 0.38–0.50) [47], both used to measure participation, and strong to very strong correlations between the PROMIS-PF and the SF-12 physical component (*r* = 0.50–0.85) [48], both used to assess physical function.

Another limitation is the relatively high percentage of missing data in both cohorts. Even though baseline data were relatively complete, more than 50% of effect outcome data were missing at 6 and 12 months in the LongCOVID cohort and at 12 months in the ParaCOV cohort. Multiple imputation is currently regarded as a valid method to handle data that is Missing at Random (MAR; i.e. the missingness of data depends on observed variables) and was therefore used in this study. Although the analysis of the imputed data yielded similar results as the complete-case analysis, there may still be bias due to missing data. Moreover, it is also possible that data were Missing Not at Random (MNAR), meaning that missingness is related to unobserved variables that could not be corrected^14^. In the current analyses, the possible effect of reinfection and vaccination after developing long-term symptoms was not explored. Lastly, our population most likely had the wild-type or Alpha variant of COVID-19, and it is unknown to what extent our results are generalizable to populations with other COVID-19 variants (e.g. Delta or Omicron) and we were not able to correct for possible re-infections during follow-up.

### Recommendations for practice and future research

Given the limitations of the current study, including its non-randomized design and hence the possible influence of unmeasured confounding, decision-makers should be careful while interpreting the results. Moreover, one should bear in mind that the current results are not necessarily transferable to other countries, as extensive differences exist across countries regarding the organization of care and the management of persistent complaints after a COVID-19 infection^17-20^. In the United Kingdom, for example, a large number of Post-COVID Assessment Clinics were already opened in 2021 that offered special physical, cognitive, and/or psychological assessments, whereas most Dutch patients were referred to allied healthcare professionals at that time [38,49].

Nevertheless, our findings may bring insights for future research on the effectiveness of rehabilitation treatment for persistent complaints after a COVID-19 infection. First, a pragmatic randomized controlled trial including more tailored rehabilitation treatments should be conducted to provide high-quality evidence on the effectiveness of these treatment programs. Second, outcome measures should be harmonized among research groups assessing (the treatment of) persistent complaints after a COVID-19 infection to ensure valid comparisons. Third, a broader scope of possible confounders should be assessed in future observational research on (the treatment of) persistent complaints after a COVID-19 infection, including infection-related factors (e.g. time since infection, severity), psychological factors (e.g. personality traits, coping mechanisms, social support), lifestyle factors (e.g. physical activity, nutrition), and physiological factors (e.g. exercise intolerance), work environmental factors (e.g. type of work, workload, workability), and socioeconomic aspects (e.g. income, catastrophic expenditures).

## Conclusions

Patients with persistent complaints after a COVID-19 infection who received allied healthcare had statistically significantly lower participation after 6 months, higher health-related quality of life after 12 months, and better physical functioning after 6 and 12 months compared with participants who did not receive allied healthcare. However, these between-group differences were not clinically relevant. Given observed effect differences between groups may reflect not only the intervention but also unmeasured confounding, decision-makers should be careful while interpreting the results. Additional research is essential to develop more tailored rehabilitation treatments and to provide robust evidence of their effectiveness in providing high-quality care for patients with persistent complaints after a COVID-19 infection.

## Supplementary Material

Supplemental Material

## Data Availability

The data that support the findings of this study are available from the corresponding author, Johanna Maria van Dongen, upon reasonable request.

## References

[1] Han Q, Zheng B, Daines L, et al. Long-term sequelae of COVID-19: a systematic review and meta-analysis of one-year follow-up studies on post-COVID symptoms. Pathogens. 2022;11(2):269. 35215212 10.3390/pathogens11020269 PMC8875269

[2] Natarajan A, Shetty A, Delanerolle G, et al. A systematic review and meta-analysis of Long COVID symptoms. 2022. March 9 [cited 2022 April 1]:2022.03.08.22272091.Available from:https://www.medrxiv.org/content/10.1101/2022.03.08.22272091v1. 10.1186/s13643-023-02250-0 PMC10220332 37245047

[3] CDC. Long COVID Basics. Long COVID [Internet]. ; 2025 August 18 [cited 2025 November 14]. Available from:https://www.cdc.gov/long-covid/about/index.html.

[4] WHO. Post COVID-19 condition (long COVID). [cited 2025 November 14]. Available from: https://www.who.int/news-room/fact-sheets/detail/post-covid-19-condition-(long-covid.)

[5] RIVM. COVID-19 Health Survey | RIVM. [cited 2024 July 16]. Available from:https://www.rivm.nl/gezondheidsonderzoek-covid-19/kwartaalonderzoek-volwassenen/lichamelijke-gezondheid.

[6] Tabacof L, Tosto-Mancuso J, Wood J, et al. Post-acute COVID-19 syndrome negatively impacts physical function, cognitive function, health-related quality of life, and participation. Am J Phys Med Rehabil. 2022;101(1):48–52. doi: 10.1097/PHM.0000000000001910. 34686631 PMC8667685

[7] Tak CR. The health impact of long COVID: a cross-sectional examination of health-related quality of life, disability, and health status among individuals with self-reported post-acute sequelae of SARS CoV-2 infection at various points of recovery. J Patient Rep Outcomes. 2023;7(1):31. doi: 10.1186/s41687-023-00572-0. 36943643 PMC10029785

[8] O’ Mahony L, Buwalda T, Blair M, et al. Impact of long COVID on health and quality of life. HRB Open Res. 2022;5:31. doi: 10.12688/hrbopenres.13516.1. 36101871 PMC9440374

[9] Wolff Sagy Y, Feldhamer I, Brammli-Greenberg S, et al. Estimating the economic burden of long-covid: the additive cost of healthcare utilisation among COVID-19 recoverees in Israel. BMJ Glob Health. 2023;8(7):e012588. doi: 10.1136/bmjgh-2023-012588. PMC10357303 37463787

[10] Cutler DM. The Costs of Long COVID. JAMA Health Forum. 2022;3(5):e221809. doi: 10.1001/jamahealthforum.2022.1809. 36219031

[11] Voruz P, Assal F, Péron JA. The economic burden of the post-COVID-19 condition: underestimated long-term consequences of neuropsychological deficits. J Glob Health. 2023;13:03019. doi: 10.7189/jogh.13.03019. 37141527 PMC10159592

[12] Gandjour A. Long COVID: costs for the German economy and health care and pension system. BMC Health Serv Res. 2023;23(1):641. doi: 10.1186/s12913-023-09601-6. 37316880 PMC10266873

[13] Ministerie van Volksgezondheid WeS. Conditional reimbursement of health care - Report - National Health Care Institute. 2012 April 6 [cited 2023 April 19]. Available from: https://english.zorginstituutnederland.nl/publications/reports/2012/04/06/conditional-reimbursement-of-health-care.

[14] Nederland Z. Aantallen gebruikers en kosten paramedische herstelzorg nemen langzaam af. [cited 2023 July 31]. Available from: https://www.zorgcijfersdatabank.nl/nieuws/covid-herstelzorg-factsheet.

[15] De Bie RA, Verburg AC, Agasi-Idenburg C, et al. Evaluation of allied healthcare in patients recovering from covid-19: study protocol and baseline data of s national prospective cohort study. J Rehabil Med. 2022;54:jrm00309. doi: 10.2340/jrm.v54.2506. 35735900 PMC9422882

[16] Slotegraaf AI, Gerards MHG, Verburg AC, et al. Evaluation of primary allied healthcare in patients recovering from COVID-19: first results after six months follow-up in a Dutch nationwide prospective cohort study - preprint. 2023 February 27 [cited 2023 July 31]:2022.10.03.22280639. Available from: https://www.medrxiv.org/content/10.1101/2022.10.03.22280639v3.

[17] Gerards MHG, Slotegraaf AI, Verburg AC, et al. One-year evaluation of people recovering from COVID-19 receiving allied primary healthcare: a nationwide prospective cohort study. Ann Phys Rehabil Med. 2024;67(7):101874. doi: 10.1016/j.rehab.2024.101874. 39173549

[18] Mutubuki EN, T van der M, Leung KY, et al. Prevalence and determinants of persistent symptoms after infection with SARS-CoV-2: Protocol for an observational cohort study (LongCOVID-study). 2022 January 11 [cited 2022 April 1]:2022.01.10.22269009. Available from: https://www.medrxiv.org/content/10.1101/2022.01.10.22269009v1. 10.1136/bmjopen-2022-062439 PMC9251892 35777877

[19] Ben ÂJ, Verburg AC, Maas ET, et al. Work trajectories of patients with persistent complaints after a COVID-19 infection receiving allied healthcare in the netherlands: a secondary analysis of the ParaCOV cohort. J Occup Environ Med. 2024;66(12):993–999. doi: 10.1097/JOM.0000000000003240. 39393926

[20] Volksgezondheid M v. Variants of the coronavirus SARS-CoV-2. The Netherlands; 2023. National Institute for Public Health and the Environment Ministry of Health, Welfare and Sport. Available from: https://www.rivm.nl/en/coronavirus-covid-19/virus/variants

[21] Worm-Smeitink M, Gielissen M, Bloot L, et al. The assessment of fatigue: psychometric qualities and norms for the checklist individual strength. J Psychosom Res. 2017;98:40–46. doi: 10.1016/j.jpsychores.2017.05.007. 28554371

[22] Snaith RP. The hospital anxiety and depression scale. Health Qual Life Outcomes. 2003;1(1):29. doi: 10.1186/1477-7525-1-29. 12914662 PMC183845

[23] Ganesh R, Ghosh AK, Nyman MA, et al. PROMIS scales for assessment of persistent post-COVID symptoms: a cross sectional study. J Prim Care Community Health. 2021;12:21501327211030413. doi: 10.1177/21501327211030413. 34231395 PMC8267017

[24] Vélez-Santamaría R, Fernández-Solana J, Méndez-López F, et al. Functionality, physical activity, fatigue and quality of life in patients with acute COVID-19 and Long COVID infection. Sci Rep. 2023;13(1):19907. doi: 10.1038/s41598-023-47218-1. 37963962 PMC10645778

[25] Schalet BD, Hays RD, Jensen SE, et al. Validity of PROMIS physical function measured in diverse clinical samples. J Clin Epidemiol. 2016;73:112–118. doi: 10.1016/j.jclinepi.2015.08.039. 26970039 PMC4968197

[26] Post MWM, van der Zee CH, Hennink J, et al. Validity of the utrecht scale for evaluation of rehabilitation-participation. Disabil Rehabil. 2012;34(6):478–485. doi: 10.3109/09638288.2011.608148. 21978031

[27] Burdine JN, Felix MR, Abel AL, et al. The SF-12 as a population health measure: an exploratory examination of potential for application. Health Serv Res. 2000;35(4):885–904. 11055454 PMC1089158

[28] Herdman M, Gudex C, Lloyd A, et al. Development and preliminary testing of the new five-level version of EQ-5D (EQ-5D-5L). Qual Life Res. 2011;20(10):1727–1736. doi: 10.1007/s11136-011-9903-x. 21479777 PMC3220807

[29] M. Versteegh M, M. Vermeulen K, M. A. A. Evers S, et al. Dutch tariff for the five-level version of EQ-5D. Value in Health. 2016;19(4):343–352. doi: 10.1016/j.jval.2016.01.003. 27325326

[30] Valko PO, Bassetti CL, Bloch KE, et al. Validation of the fatigue severity scale in a swiss cohort. Sleep. 2008;31(11):1601–1607. doi: 10.1093/sleep/31.11.1601. 19014080 PMC2579971

[31] Terwee CB, Roorda LD, de Vet HCW, et al. Dutch–flemish translation of 17 item banks from the patient-reported outcomes measurement information system (PROMIS). Qual Life Res. 2014;23(6):1733–1741. doi: 10.1007/s11136-013-0611-6. 24402179

[32] Ware J, Kosinski M, Keller SD. A 12-Item short-form health survey: construction of scales and preliminary tests of reliability and validity. Med Care. 1996;34(3):220–233. doi: 10.1097/00005650-199603000-00003. 8628042

[33] Vaishnav AS, Gang CH, Iyer S, et al. Correlation between NDI, PROMIS and SF-12 in cervical spine surgery. Spine J. 2020;20(3):409–416. doi: 10.1016/j.spinee.2019.10.017. 31678044 PMC11620278

[34] White IR, Royston P, Wood AM. Multiple imputation using chained equations: issues and guidance for practice. Stat Med. 2011;30(4):377–399. doi: 10.1002/sim.4067. 21225900

[35] Kreif N, Grieve R, Radice R, et al. Regression-adjusted matching and double-robust methods for estimating average treatment effects in health economic evaluation. Health Serv Outcomes Res Method. 2013;13(2-4):174–202. doi: 10.1007/s10742-013-0109-2.

[36] Markoulidakis A, Taiyari K, Holmans P, et al. A tutorial comparing different covariate balancing methods with an application evaluating the causal effects of substance use treatment programs for adolescents. Health Serv Outcomes Res Methodol. 2023;23(2):115–148. doi: 10.1007/s10742-022-00280-0. 37207016 PMC10188586

[37] Henry EB, Barry LE, Hobbins AP, et al. Estimation of an instrument-defined minimally important difference in EQ-5D-5L index scores based on scoring algorithms derived using the EQ-VT Version 2 valuation protocols. Value Health. 2020;23(7):936–944. doi: 10.1016/j.jval.2020.03.003. 32762996

[38] NHS. Coronavirus » Allied health professionals’ role in rehabilitation during and after COVID-19. [cited 2023 August 11]. Available from: https://www.england.nhs.uk/coronavirus/documents/allied-health-professionals-role-in-rehabilitation-during-and-after-covid-19/.

[39] Sánchez-García JC, Reinoso-Cobo A, Piqueras-Sola B, et al. Long COVID and physical therapy: A systematic review. Diseases. 2023;11(4):163. doi: 10.3390/diseases11040163. 37987274 PMC10660729

[40] Jimeno-Almazán A, Franco-López F, Buendía-Romero Á, et al. Rehabilitation for post-COVID-19 condition through a supervised exercise intervention: A randomized controlled trial. Scand J Med Sci Sports. 2022;32(12):1791–1801. doi: 10.1111/sms.14240. 36111386 PMC9538729

[41] Philip KEJ, Owles H, McVey S, et al. An online breathing and wellbeing programme (ENO Breathe) for people with persistent symptoms following COVID-19: a parallel-group, single-blind, randomised controlled trial. Lancet Respir Med. 2022;10(9):851–862. doi: 10.1016/S2213-2600(22)00125-4. 35489367 PMC9045747

[42] McGregor G, Sandhu H, Bruce J, et al. Clinical effectiveness of an online supervised group physical and mental health rehabilitation programme for adults with post-covid-19 condition (REGAIN study): multicentre randomised controlled trial. BMJ. 2024;384:e076506. doi: 10.1136/bmj-2023-076506. 38325873 PMC11134408

[43] Ferreira ML, Herbert RD, Ferreira PH, et al. The smallest worthwhile effect of nonsteroidal anti-inflammatory drugs and physiotherapy for chronic low back pain: a benefit-harm trade-off study. J Clin Epidemiol. 2013;66(12):1397–1404. doi: 10.1016/j.jclinepi.2013.02.018. 24021611

[44] Christiansen DH, de Vos Andersen N-B, Poulsen PH, et al. The smallest worthwhile effect of primary care physiotherapy did not differ across musculoskeletal pain sites. J Clin Epidemiol. 2018;101:44–52. doi: 10.1016/j.jclinepi.2018.05.019. 29852251

[45] Gruber S, van der Laan MJ. A targeted maximum likelihood estimator of a causal effect on a bounded continuous outcome. Int J Biostat. 2010;6(1):Article 26. doi: 10.2202/1557-4679.1260. 21731529 PMC3126669

[46] Varga AN, Guevara Morel AE, Lokkerbol J, et al. Dealing with confounding in observational studies: A scoping review of methods evaluated in simulation studies with single-point exposure. Stat Med. 2023;42(4):487–516. doi: 10.1002/sim.9628. 36562408 PMC10107671

[47] Lee J-H, Park J-H, Kim YJ, et al. Validity and reliability of the korean version of the utrecht scale for evaluation of rehabilitation-participation. Occup Ther Int. 2017;2017:9452051–9452055. doi: 10.1155/2017/9452051. 29097981 PMC5612671

[48] Young K, Steinhaus M, Gang C, et al. The use of patient-reported outcomes measurement information system in spine: a systematic review. Int J Spine Surg. 2021;15(1):186–194. doi: 10.14444/8024. 33900973 PMC7931740

[49] NHS. Statistics ≫ COVID-19 Post-covid assessment service. [cited 2024 May 24]. Available from: https://www.england.nhs.uk/statistics/statistical-work-areas/covid-19-post-covid-assessment-service/.

