# Supplementary material for: A comparison of allied healthcare versus no allied healthcare on participation, fatigue, physical functioning and health-related quality of life for patients with persistent complaints after a COVID-19 infection
Source: Ann Med. 2025 Dec 10;57(1):2600139. doi: 10.1080/07853890.2025.2600139 (PMC12720634; doi:10.1080/07853890.2025.2600139)
Supplement: Supplemental Material [file IANN_A_2600139_SM6502.zip › suppl_data/appendix c.docx]

**APPENDIX C. MISSING DATA – DESCRIPTIVE ANALYSIS ORIGINAL DATA**

| **Allied healthcare (yes/no)** | | **N** | **Mean** | **Standard Deviation** | **Missing** | |
| --- | --- | --- | --- | --- | --- | --- |
|  |  |  |  |  | **Count** | **Percent** |
| **Baseline characteristics** | | | | | | |
| No | Age | 1427 | 41.4 | 13.7 | 0 | 0 |
|  | Sex | 1427 |  |  | 0 | 0 |
|  | BMI | 1401 | 26.1 | 4.9 | 26 | 1.8 |
|  | Smoking status | 1325 |  |  | 102 | 7.1 |
|  | Comorbidities | 1427 | 11.9 | 2.5 | 0 | 0 |
|  | HADS-A | 1427 | 9.3 | 1.9 | 0 | 0 |
|  | HADS_D | 1427 |  |  | 0 | 0 |
| Yes | Age | 1313 | 49.1 | 12.9 | 138 | 9.5 |
|  | Sex | 1312 |  |  | 139 | 9.6 |
|  | BMI | 1039 | 28.2 | 5.7 | 412 | 28.4 |
|  | Smoking status | 1287 |  |  | 164 | 11.3 |
|  | Comorbidities | 1313 |  |  | 138 | 9.5 |
|  | HADS-A | 1278 | 7.1 | 4.5 | 173 | 11.9 |
|  | HADS_D | 1278 | 7.3 | 4.2 | 173 | 11.9 |
| **Effect outcomes** | | | | | | |
| No | Participation (baseline) | 1,427 | 68.31 | 31.86 | 0 | 0.0 |
|  | Participation (6-month) | 541 | 83.53 | 24.87 | 886 | 62.1 |
|  | Participation (12-month) | 380 | 83.68 | 24.77 | 1047 | 73.4 |
| Yes | Participation (baseline) | 1,278 | 65.78 | 19.28 | 173 | 11.9 |
|  | Participation (6-month) | 926 | 78.21 | 18.97 | 525 | 36.2 |
|  | Participation (12-month) | 677 | 79.81 | 18.88 | 774 | 53.3 |
|  | | | | | | |
| No | Health-related quality of life (baseline) | 1,427 | 0.727 | 0.202 | 0 | 0.0 |
|  | Health-related quality of life (6-month) | 541 | 0.805 | 0.186 | 886 | 62.1 |
|  | Health-related quality of life (12-month) | 380 | 0.797 | 0.204 | 1047 | 73.4 |
| Yes | Health-related quality of life (baseline) | 1,304 | 0.628 | 0.220 | 147 | 10.1 |
|  | Health-related quality of life (6-month) | 940 | 0.742 | 0.214 | 511 | 35.2 |
|  | Health-related quality of life (12-month) | 739 | 0.756 | 0.218 | 712 | 49.1 |
|  | | | | | | |
| No | Fatigue (baseline) | 1,427 | 75.94 | 16.80 | 0 | 0.0 |
|  | Fatigue (6-month) | 541 | 60.92 | 22.77 | 886 | 62.1 |
|  | Fatigue (12-month) | 380 | 60.25 | 23.13 | 1047 | 73.4 |
| Yes | Fatigue (baseline) | 1,294 | 76.76 | 16.39 | 157 | 10.8 |
|  | Fatigue (6-month) | 936 | 65.54 | 21.68 | 515 | 35.5 |
|  | Fatigue (12-month) | 690 | 64.81 | 22.71 | 761 | 52.4 |
|  | | | | | | |
| No | Physical Functioning (baseline) | 1,427 | 61.01 | 21.38 | 0 | 0.0 |
|  | Physical Functioning (6-month) | 487 | 71.22 | 17.89 | 940 | 65.9 |
|  | Physical Functioning (12-month) | 330 | 71.53 | 19.13 | 1097 | 76.9 |
| Yes | Physical Functioning (baseline) | 1,286 | 59.62 | 19.73 | 165 | 11.4 |
|  | Physical Functioning (6-month) | 936 | 74.16 | 20.64 | 515 | 35.5 |
|  | Physical Functioning (12-month) | 687 | 74.64 | 21.36 | 764 | 52.7 |
