# Supplementary material for: A comparison of allied healthcare versus no allied healthcare on participation, fatigue, physical functioning and health-related quality of life for patients with persistent complaints after a COVID-19 infection
Source: Ann Med. 2025 Dec 10;57(1):2600139. doi: 10.1080/07853890.2025.2600139 (PMC12720634; doi:10.1080/07853890.2025.2600139)
Supplement: Supplemental Material [file IANN_A_2600139_SM6502.zip › suppl_data/appendix d.docx]

**APPENDIX D. BASELINE CHARACTERISTICS OF PARTICIPANTS WHO RECEIVED ALLIED HEALTHCARE IN BOTH COHORTS**

| **Baseline characteristic** | **Allied healthcare**  ***n = 1,451***  **(ParaCOV)** | **LongCOVID with allied healthcare**  ***n = 3,466*** | **p-value** |
| --- | --- | --- | --- |
| Age, mean (SE) | 49.1 (0.4) | 45.4 (0.2) | 0.001 |
| Female, n (%) | 834 (63.6) | 2823 (81.4) | 0.001 |
| BMI, mean (SE) | 28.2 (0.2) | 27.0 (0.1) | 0.001 |
| Smoking status |  |  |  |
| Never/ former | 1361 (95.0) | 3198 (94.8) | 0.607 |
| Current | 71 (5.0) | 175 (5.2) |  |
| Comorbidities, n (%) |  |  |  |
| None | 727 (50.8) | 1724 (49.7) | 0.252 |
| ≥ 1 comorbidity | 705 (49.2) | 1742 (50.3) |  |
| HADS-A, mean (SE) | 7.0 (0.1) | 11.8 (0.04) | 0.001 |
| HADS-D, mean (SE) | 7.3 (0.1) | 9.4 (0.03) | 0.001 |
| Participation, mean (SE) | 65.8 (0.5) | 46.4 (0.6) | 0.001 |
| Health-related quality of life, mean (SE) | 0.628 (0.006) | 0.572 (0.004) | 0.001 |
| Fatigue, mean (SE) | 76.8 (0.4) | 81.0 (0.3) | 0.001 |
| Physical functioning, mean (SE) | 59.6 (0.5) | 43.9 (0.4) | 0.001 |

SE = standard error. n = number of participants. % = proportion. BMI = Body Mass Index. Comorbidities included hypertension, diabetes mellitus, chronic obstructive pulmonary disease, kidney disease, liver disease, immune disease, oncological disease/malignancies, and chronic neuromuscular disorders. HADS-A = Hospital Anxiety and Depression Scale possible anxiety. HADS-D = Hospital Anxiety and Depression Scale possible depression. HADS scores ≥ 11 indicate a high probability of anxiety or depression. Participation was measured by the Utrecht Scale for Evaluation of Rehabilitation- Participation (USER-P) subscale restrictions in the ParaCOV study, while in the LongCOVID study, it was measured by the SF-12 Role Physical Limitation questions, both rescaled to 0-100, high scores represent high participation. Health-related quality of life was measured by the EQ-5D-5L; the Dutch EQ-5D-5L tariffs range from -0.446 to 1 (full health). Fatigue was measured by the Fatigue Severity Scale (FSS) in the ParaCOV study while in the LongCOVID study, it was measured by the Checklist Individual Strength (CIS), both rescaled to 0-100, high scores represent more fatigue. Physical functioning was measured by the Patient-Reported Outcomes Measurement Information System Physical Functioning Short Form 10b (PROMIS-PF-10b) in the ParaCOV study while in the LongCOVID study, measured by the SF-12 Physical Component Summary, both rescaled to 0-100, high scores represent better physical functioning.
