# Supplementary material for: A comparison of allied healthcare versus no allied healthcare on participation, fatigue, physical functioning and health-related quality of life for patients with persistent complaints after a COVID-19 infection
Source: Ann Med. 2025 Dec 10;57(1):2600139. doi: 10.1080/07853890.2025.2600139 (PMC12720634; doi:10.1080/07853890.2025.2600139)
Supplement: Supplemental Material [file IANN_A_2600139_SM6502.zip › suppl_data/appendix e.docx]

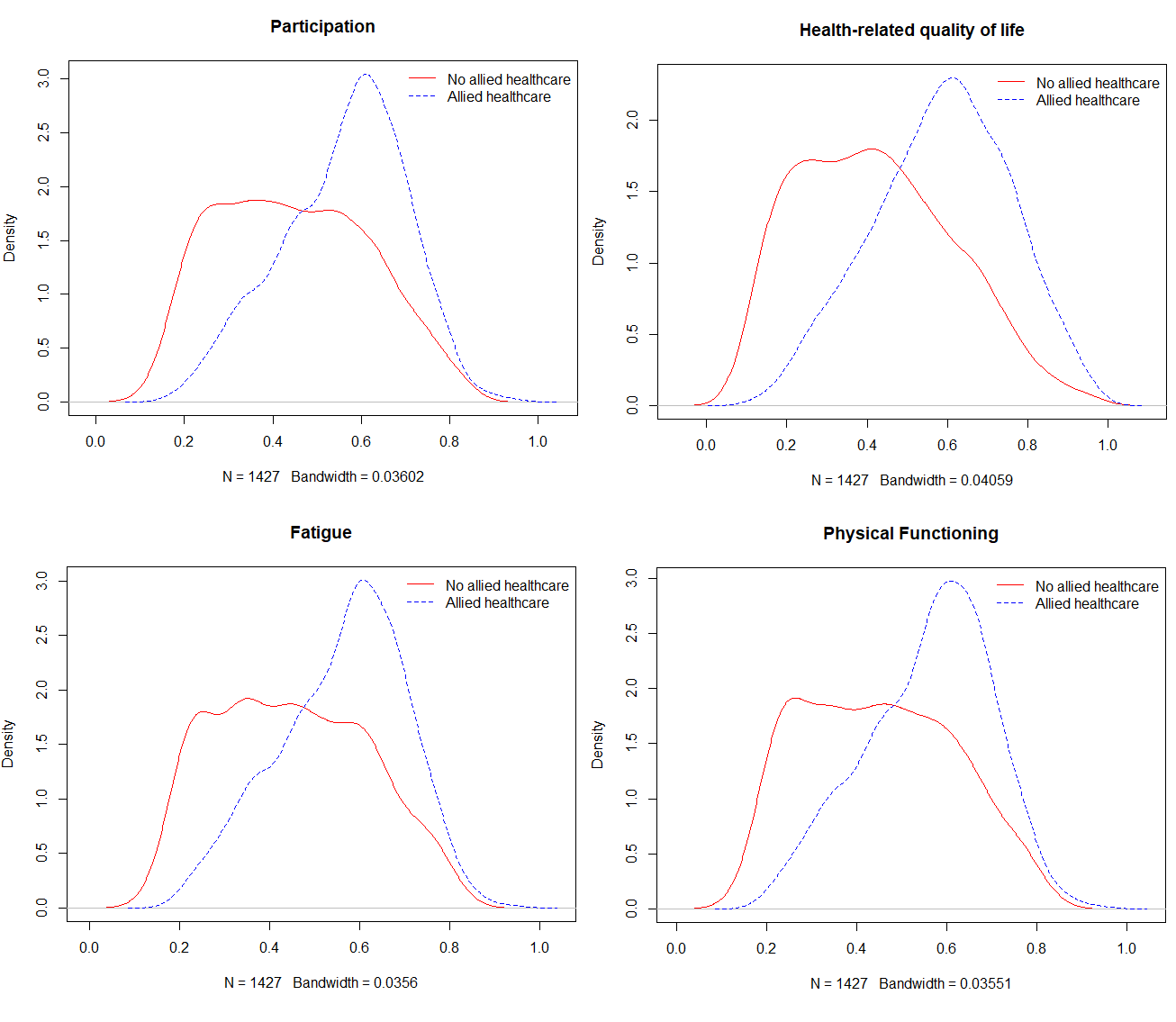


**Appendix E.** Density plots of intervention and control show a relatively good overlap of the propensity score distributions between the two groups when adjusting effect outcomes for their respective baseline values, age, sex, body mass index, smoking status, and comorbidities. A good overlap between the propensity scores of the control and intervention groups means that the two groups are more comparable. This is relevant because it helps ensure that any differences observed between the groups are likely due to the intervention itself and not because the groups were initially different.
