# Supplementary material for: A comparison of allied healthcare versus no allied healthcare on participation, fatigue, physical functioning and health-related quality of life for patients with persistent complaints after a COVID-19 infection
Source: Ann Med. 2025 Dec 10;57(1):2600139. doi: 10.1080/07853890.2025.2600139 (PMC12720634; doi:10.1080/07853890.2025.2600139)
Supplement: Supplemental Material [file IANN_A_2600139_SM6502.zip › suppl_data/appendix f.docx]

**
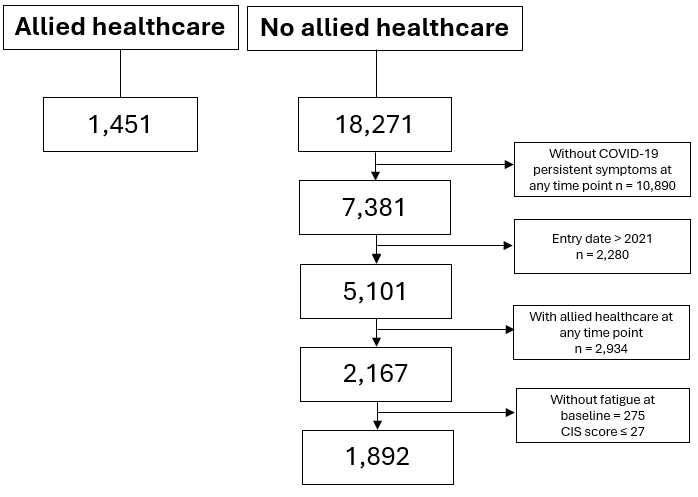
**

**Appendix F.** Flowchart showing the study population from the ParaCOV cohort and the LongCOVID cohort according to eligibility criteria but including, in the no allied healthcare group, participants from the LongCOVID cohort who received allied healthcare but unrelated to a COVID-19 infection (i.e., sensitivity analysis 2). CIS = Checklist Individual Strength. The mean CIS score ≤27 was used as a cut-off point to ensure comparability between both cohorts in terms of fatigue at baseline. NB: Please note that we only received LongCOVID data of adult people with a self-reported positive SARS-CoV-2 test.
